# The Role of Matrix Gla Protein (MGP) Expression in Paclitaxel and Topotecan Resistant Ovarian Cancer Cell Lines

**DOI:** 10.3390/ijms19102901

**Published:** 2018-09-25

**Authors:** Karolina Sterzyńska, Andrzej Klejewski, Karolina Wojtowicz, Monika Świerczewska, Małgorzata Andrzejewska, Damian Rusek, Maciej Sobkowski, Witold Kędzia, Jacek Brązert, Michał Nowicki, Radosław Januchowski

**Affiliations:** 1 Department of Histology and Embryology, Poznan University of Medical Sciences, Święcickiego 6 St., 61-781 Poznań, Poland; kwojtowicz@ump.edu.pl (K.W.); mswierczewska@ump.edu.pl (M.S.); mandrzej@ump.edu.pl (M.A.); mnowicki@ump.edu.pl (M.N.); rjanuchowski@ump.edu.pl (R.J.); 2 Department of Nursing, Poznan University of Medical Sciences, Smoluchowskiego 11 St., 60-179 Poznań, Poland; aklejewski@ump.edu.pl; 3 Department of Obstetrics and Women’s Diseases, Poznan University of Medical Sciences, Polna 33 St, 60-535 Poznań, Poland; jbrazert@ump.edu.pl; 4 Department of Pathomorphology, Non-public Health Care Facility Alfamed, Jana Pawła II 10 St, 22-400 Zamość, Poland; d.rusek@vp.pl; 5 Department of Mother and Child Health, Poznan University of Medical Sciences, Polna 33 St, 60-535 Poznań, Poland; msobkow@ump.edu.pl; 6 Department of Gynecology, Poznan University of Medical Sciences, Polna 33 St, 60-535 Poznań, Poland; witold.kedzia@ump.edu.pl

**Keywords:** matrix Gla protein, ovarian cancer, paclitaxel resistance, topotecan resistance

## Abstract

The major cause of ovarian cancer treatment failure in cancer patients is inherent or acquired during treatment drug resistance of cancer. Matrix Gla protein (MGP) is a secreted, non-collagenous extracellular matrix protein involved in inhibition of tissue calcification. Recently, MGP expression was related to cellular differentiation and tumor progression. A detailed MGP expression analysis in sensitive (A2780) and resistant to paclitaxel (PAC) (A2780PR) and topotecan (TOP) (A2780TR) ovarian cancer cell lines and their corresponding media was performed. *MGP* mRNA level (real time PCR analysis) and protein expression in cell lysates and cell culture medium (Western blot analysis) and protein expression in cancer cells (immunofluorescence analysis) and cancer patient lesions (immunohistochemistry) were determined in this study. We observed increased expression of MGP in PAC and TOP resistant cell lines at both mRNA and protein level. MGP protein was also detected in the corresponding culture media. Finally, we detected expression of MGP protein in ovarian cancer lesions from different histological type of cancer. MGP is an important factor that might contribute to cancer resistance mechanism by augmenting the interaction of cells with ECM components leading to increased resistance of ovarian cancer cells to paclitaxel and topotecan. Expression found in ovarian cancer tissue suggests its possible role in ovarian cancer pathogenesis.

## 1. Introduction

The major cause of ovarian cancer treatment failure is tumoral heterogeneity and occurrence of drug resistance in ovarian cancer patients. There are two mechanisms of inherent or acquired during treatment chemoresistance that involve cellular and/or tissue specific response. The cellular mechanisms include reduced drug accumulation and changes in drug distribution in the cell, faster inactivation of the drug and more efficient mechanisms of DNA and cellular membranes repair caused by the drug [1]. However, one of the principal mechanisms of cellular chemoresistance is multiple drug resistance (MDR). The cancer cell acquires the ability to actively pump cytotoxic drugs out of the cell via drug transporters. The best known are ABC family transporters, with glycoprotein P (P-gp) and BCRP (breast cancer resistant protein) as the most extensively studied ones [2,3] Those proteins facilitate the outflow of chemotherapeutic agents and thereby contribute significantly to cancer cell resistance. On the other hand, the knowledge concerning a cancer tissue specific mechanism of chemoresistance is still deficient. The tumor environment is now identified as a leading factor that promotes cancer progression and resistance to anticancer therapy [4]. It is composed of cellular (cancer cells, cancer associated fibroblasts (CAFs), and tumor associated macrophages (TAMs)) and non-cellular constituents including extracellular matrix components (ECM). Increased expression of proteoglycans or collagens in tumor microenvironment create a barrier and inhibits the diffusion of anti-cancer agents [5]. It has been reported that changes in ECM can modulate drug resistance by preventing the penetration of anticancer drugs through tumor tissue [6,7]. Changes in the microenvironment induce tumor progression, malignancy and anticancer drug resistance through integrin signaling as the result of ECM tumor-associated remodeling [8]. Cancer cells show decreased sensitivity to apoptosis influenced by ECM components that leads to induction of their resistance [9]. The development of drug resistance caused by compounds of extracellular matrix has been described as cell adhesion-mediated drug resistance (CAM-DR) [10] and noted in small cell lung cancer in vivo and in ovarian cancer in vitro [11,12] However, different molecules of ECM have also been described as produced by cultured cells in vitro, e.g., drug resistant breast [13] and ovarian cancer cell lines [14,15].

Matrix Gla protein (MGP) is a secreted, non-collagenous extracellular matrix protein containing post-translationally modified γ-carboxyglutamic acid residues resulting from vitamin K-dependent carboxylation [16]. The protein was initially isolated from bone and cartilage [17,18] but in human it is found also in soft tissues such as kidney, lung, heart and vascular smooth muscle cells [19]. The physiological role of this protein is to inhibit tissue calcification [20], but it is also implicated in pathological calcifications [21] as well as abnormal angiogenesis that can promote tumor progression [22]. It has been additionally suggested that MGP expression is related to cellular differentiation and tumor progression [23,24]. However, MGP expression may be tumor type dependent. On the one hand, there is a negative correlation of MGP expression with tumor progression and metastasis in renal and prostate carcinoma [25]. On the other hand, up-regulation of MGP transcript in breast tumors and glioblastomas is associated with tumor progression and poor prognosis [24,26].

Little is known about the mechanisms linking upregulation of MGP with migration ability, metastases formation or drug resistance, and, more generally, the function of MGP in tumors. Recently, it was proved that MGP binds fibronectin and augments cell adhesion and spreading of cancer cells [27]. Increased expression of MGP in glioblastoma cells is associated with increased tumor cells migratory properties in vitro and tumor spreading in vivo, whereas MGP-knock down leads to opposite effect [26]. Overexpression of MGP in osteosarcoma cells leads to statistically significant number and size of lung metastasis in mouse model. Furthermore, MGP serum level is associated with lung metastasis in osteosarcoma patients [28].

To gain a better understanding of the role of MGP in drug resistance development, we used an ovarian cancer model, which is the most lethal gynecological malignancy [29]. Although ovarian cancer is sensitive to chemotherapy, especially at early stages, most patients will eventually develop resistance to anti-cancer drugs. The first-line chemotherapy regimen for ovarian cancer treatment combine chemotherapy of platinum (cisplatin (CIS)) and taxanes (paclitaxel (PAC)) [29]. The second line chemotherapy includes doxorubicin (DOX) and topotecan (TOP), among others [30].

In this study, we used two different cell lines resistant to PAC and TOP. Paclitaxel belongs to the family of antimitotic anticancer agents that block mitosis through stabilizing microtubules, which consequently prevents cell division and leads to apoptotic cell death [31]. On the other hand, topotecan is semi-synthetic derivative of camptothecin [32] and acts as an inhibitor of DNA topoisomerase I. Inhibition of DNA topoisomerase I results in inhibition of DNA replication and transcription that eventually leads to cancer cell death [33].

Unfortunately, cancer cells can develop resistance to both cytotoxic agents. The mechanism of resistance to PAC and TOP is related to altered expression of β-tubulin [34] or Topoizomerase I [35], respectively. However, it is considered that the most important mechanism responsible for the resistance to PAC and TOP is the overexpression of drug transporters from ABC family [3,36]. 

The results of our previous microarray tests indicated that MGP was overexpressed in five of eight drug-resistant ovarian cancer cell lines [37]. In this study, we performed detailed expression analysis of MGP at mRNA and protein levels in sensitive (A2780) and resistant to paclitaxel (A2780PR1) and topotecan (A2780TR1 and A2780TR2) ovarian cancer cell lines and their corresponding media. Additionally, analysis of paraffin sections confirmed the presence of MGP in ovarian cancer tissue.

## 2. Results

### 2.1. Analyses of MGP Gene Expression in Drug-Resistant Ovarian Cancer and Breast Cancer Cell Lines

**Figure 1:**
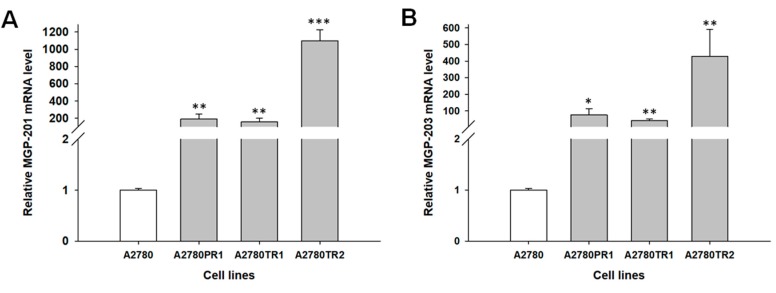
Expression analysis (Q-PCR) of the *MGP1-201* (**A**) and MGP-203 (**B**) transcripts in the A2780 and drug resistant sublines. The figure presents the relative gene expression in the resistant cell lines (grey bars) with respect to that in the sensitive cell line (white bars), which was assigned a value of 1. The values were considered significant at * *p <* 0.05, ** *p <* 0.01 and *** *p* < 0.001.

According the ENSEMBL database (www.ensembl.org), MGP is expressed in two main transcript variants (Table 1) corresponding to two protein isoforms with molecular weight of 15.32 kDa (128 aa) (*MGP-201*) and molecular weight of 12.35 kDa (103 aa) (*MGP-203*). To determine expression of both transcripts, we designed two pairs of primers that specifically recognize both variants (Table 1). To determine whether the development of drug resistance in A2780 drug-resistant sublines is associated with *MGP* overexpression, expression of the *MGP* mRNAs was assessed. We observed a statistically significant increase of both *MGP* transcripts in A2780PR1 (*p <* 0.01 for MGP-201 and *p* < 0.05 for MGP-203), A2780TR1 (*p <* 0.01 for MGP-201 and *p* < 0.01 for MGP-203) and A2780TR2 cell lines (*p <* 0.001 for MGP-201 and *p* < 0.01 for MGP-203) (Figure 1A,B). We observed proportional increase of both transcripts level in investigated cell lines (R^2^ = 0.998), however the expression of *MGP-201* was higher than *MGP-203* in all resistant cell lines; 189- vs. 77-fold (about 2.5-fold) for A2780PR1, 155- vs. 43-fold (about 3.5-fold) for A2780TR1 and 1098- vs. 428-fold (about 2.5–fold) for A2780TR2 cell line.

**Figure 2:**
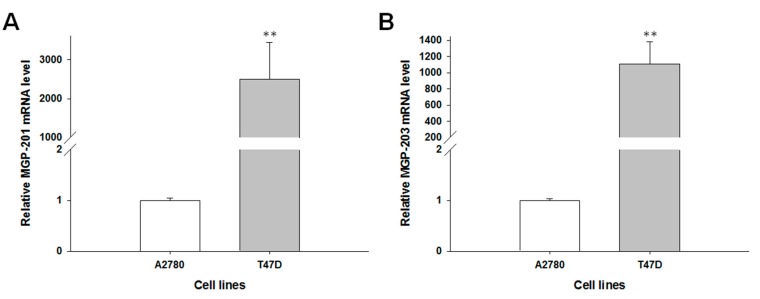
Expression analysis (Q-PCR) of the *MGP1-201* (**A**) and MGP-203 (**B**) transcripts. The figure presents the relative gene expression in the breast cancer cell line T47D (grey bars) with respect to that in the control ovarian cancer cell line A2780 (white bars), which was assigned a value of 1. The values were considered significant at ** *p <* 0.01.

To check if both *MGP* transcripts are also present in another cancer cell line, we compared their expression between A2780 cell line and breast cancer cell T47D, which is known to express *MGP* at very high level [38]. We observed statistically significant increase in expression of both transcripts in T47 cell line. Here, we also observed higher increase of *MGP-201* transcript (about 2500-fold, *p* < 0.01) than *MGP-203* (about 1100-fold, *p* < 0.01) (Figure 2A,B). However, in comparison to control A2780 cell line, increase in both transcripts level was higher in T47D cell line than in A2780T2 cell line (*MGP-201,* 2500-fold vs. 1098-fold; *MGP-203,* 1100-fold vs. 428-fold).

### 2.2. Immunofluorescence Analysis of the MGP Protein Expression

**Figure 3:**
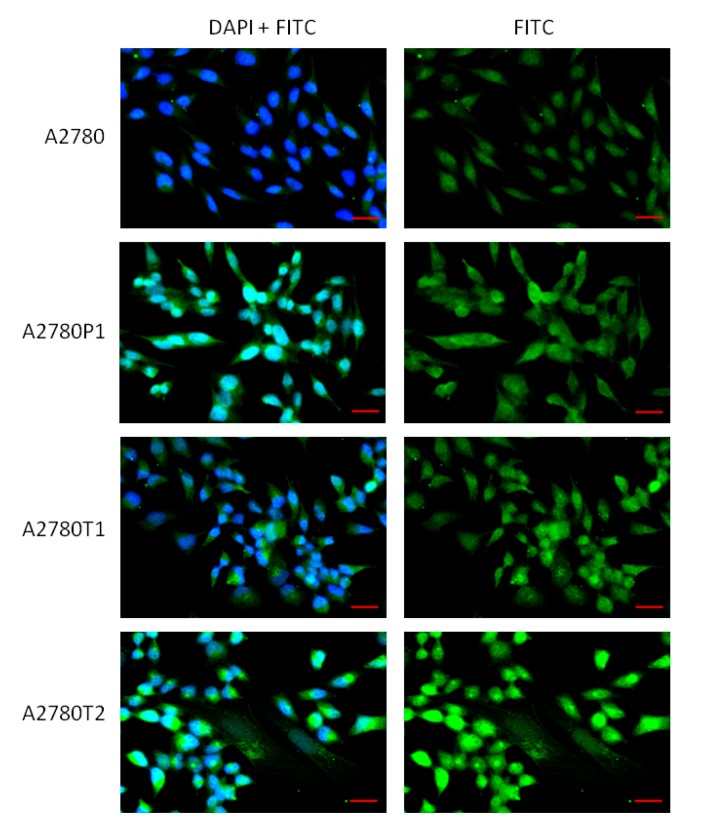
Immunofluorescence visualization of MGP protein expression in the A2780, A2780PR1, A2780TR1, A2780TR2 cell lines. MGP was detected using the anti-MGP antibody and MFP488-conjugated secondary antibody (green). To visualize the cell nuclei, the cells were mounted with a DAPI-containing mounting medium (blue). Scale bar = 20 μm.

To confirm the presence of the MGP protein in the investigated cell lines, we performed fluorescence analysis of its expression in A2780, A2780PR1, A2780TR1 and A2780TR2 cell lines. A low fluorescence signal was present in the A2780 cell line. In the A2780PR1, A2780TR1, and A2780TR2 cell lines, we observed increase in fluorescence intensity (Figure 3).

### 2.3. Western Blot Analysis of MGP Protein Expression

**Figure 4:**
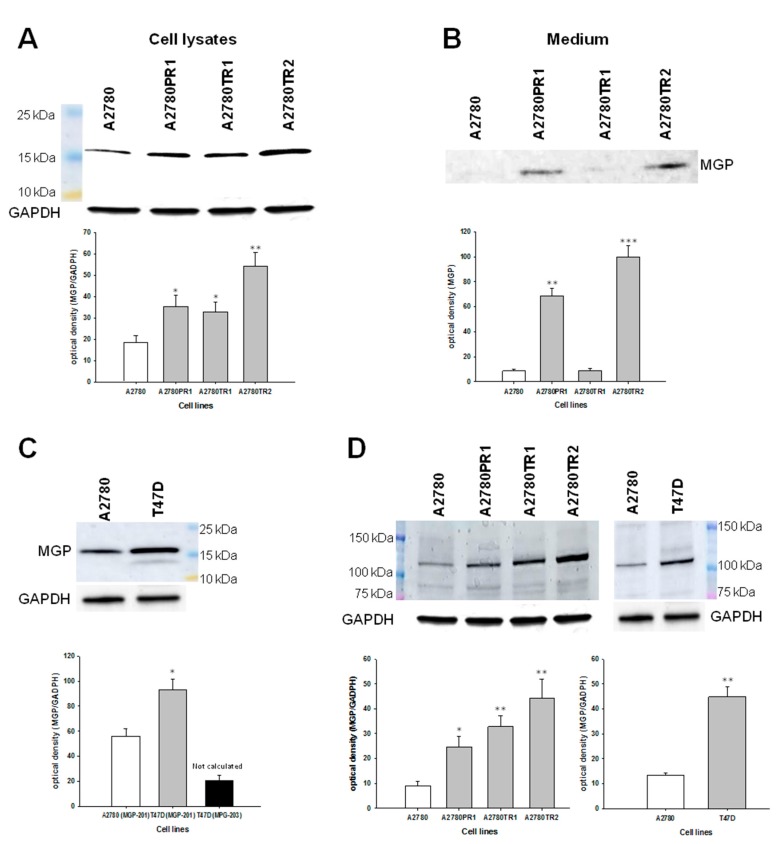
MGP protein expression analysis: in the A2780 and drug-resistant cell lines (**A**); in the corresponding media (**B**); in the A2780 and T47D cell lines (**C**); and analysis of band with high molecular mass in all investigated cell lines (**D**). The cellular proteins were separated using 7% PAGE and transferred to a PVDF membrane, which was then immunoblotted with either primary Ab or HRP-conjugated secondary Ab. A primary anti-GADPH Ab was used as a loading control for the cell lysates. The graphs show the results of the densitometric quantification of the Western blot analysis optical density, which is presented as a MGP/GADPH ratio (with exception of (**B**), presenting MGP optical density). The values were considered significant at * *p* < 0.05, ** *p* < 0.01 and *** *p* < 0.001.

The elevated expression of MGP at the protein level was confirmed by Western blot analysis. In cell lysates, we observed increase in MGP bands intensity in both PAC- (*p* < 0.05) and TOP-resistant A2780 cell lines (*p* < 0.05 for A2780TR1 and *p* < 0.01 for A2780TR2). However, we observed only one band corresponding to molecular mass of 15.32 kDa (Figure 4A).

In contrast in the corresponding medium we observed band with mass of 12.35 kDa. This band was clearly present in A2780PR1 and A2780TR2 cell lines and very weak signal was observed in A2780TR1 cell line and signal was not present in A2780 cell line (Figure 4B). Next, we analyzed expression of MGP protein in T47D cell line. Clear increase in band intensity with molecular weight of 15.32 kDa was observed in T47D cell line in comparison to A2780 cell line (*p* < 0.05). In T47D cell line, we also observed additional band corresponding to molecular weight of 12.35 kDa, although the intensity of this band was very low (Figure 4C). Additionally, in all lysates of investigated cell lines we observed additional band with molecular mass about 110 kDa. Bands intensity correlated with MGP transcripts level in all investigated cell lines (Figure 4D).

### 2.4. Early Response to PAC and TOP Treatment in Ovarian Cancer Cell Lines

**Figure 5:**
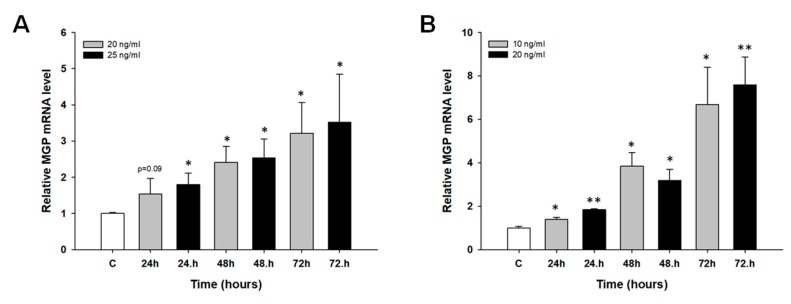
Expression analysis of *MGP* gene in A2780 cell line after short time exposure to PAC (**A**) and TOP (**B**). The figure presents relative genes expression in PAC or TOP treated cells (grey and black bars) with respect to the untreated control (white bars) assigned as 1. The values were considered significant at * *p <* 0.05, and ** *p* < 0.01.

The second part of our study focused on the early response to PAC and TOP treatment. In these experiments, drug-sensitive cell line A2780 was treated with low concentrations of PAC (20 and 25 ng/mL) or low concentration of TOP (10 and 20 ng/mL) for 24, 48 and 72 h. Then, changes in gene expression were investigated. We used primers that recognize both MGP transcript variants. We observed dose and time dependent increase in *MGP* transcript level (*p* < 0.05 with exception of 20 ng/mL and 24 h) in response to PAC treatment with maximum increase in transcript level—about 3.5-fold—after 72 h of treatment (Figure 5A). Similar time dependent increase in *MGP* transcript level was observed after TOP treatment (*p* < 0.05 or *p* < 0.01) with maximum increase in transcript level—about 8-fold—after 72 h of treatment (Figure 5B). 

### 2.5. Immunohistochemistry

**Figure 6:**
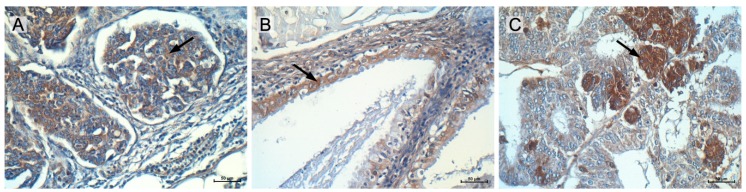
Immunohistochemical expression of MGP in serous adenocarcinoma patient (weak) (**A**); mucinous ovarian cancer patient (weak) (**B**); and endometrioid adenocarcinoma patient (moderate/strong) (**C**). Arrows point the cytoplasmic MGP expression in cancer cells. Sections were counterstained with hematoxylin. Scale bar = 50 μm.

Immunohistochemical analysis of the MGP protein was performed in ovarian cancer patients. The purpose of this analysis was to verify whether the expression of the analyzed MGP gene and protein that was observed in cell lines could also be confirmed in real cancer patient tissues. Few cases of endometrioid, serous and mucinous ovarian cancer was analyzed to determine differences in MGP expression in various subtypes of cancer. All ovarian cancer subtypes cells expressed MGP, with lower expression in serous and mucinous carcinoma (mild IRS score) (Figure 6A,B) when compared to endometrioid adenocarcinoma (moderate IRS score) (Figure 6C). Additionally, in the endometrioid adenocarcinoma, we could observe two subpopulations of cancer cells with different levels of MGP protein expression (high and weak/moderate). There was no difference in MGP expression in stroma of analyzed specimens, where all cancer subtypes showed weak expression. 

## 3. Discussion

The most significant problem with low efficiency of chemotherapy in cancer patients results from development of drug resistance to cytotoxic agents. For many years, most research has focused on cellular mechanisms of chemoresistance. However, an increasing body of evidence indicates that expression of ECM proteins in the tumor environment can play even more important role in drug resistance, especially from the clinical point of view [10,39]. It is worth mentioning that expression of ECM and related proteins is limited not only to tumor stroma and cancer-associated fibroblasts (CAF) but was also observed in tumor cells in vivo [10,12,15,40,41] and in cancer cell lines [13,37,42].

Recently, with the use of the RNA microarray technique, we have identified many new genes with increased expression in drug resistant cell lines [37]. Among them, we identified matrix Gla protein (MGP) with elevated expression levels in ovarian cancer cell lines resistant to PAC and TOP. Because nothing is known about the mechanisms linking upregulation of MGP and resistance to chemotherapeutic drugs and, more generally, about the function of MGP in ovarian cancer, we hypothesized that MGP promotes ovarian cancer progression and resistance to chemotherapy. Therefore, we decided to investigate the expression profile of MGP in PAC- and TOP-resistant ovarian cancer cell lines in more detail.

Since MGP is expressed in two main transcript variants corresponding to two protein isoforms with molecular weight of 15.32 kDa (128 aa) (*MGP-201*) and 12.35 kDa (103 aa) (*MGP-203*), we compared the expression levels of both MGP transcripts in ovarian cancer drug sensitive and resistant cell lines. We observed high increase in expression of *MGP* in the cell lines resistant to PAC (A2780PR1) and TOP (A2780TR1 and A2780TR2), with the highest level for A2780TR2 cell line. Both transcript variants were upregulated in drug resistant sublines although expression of *MGP-201* was always higher (2.5–3.5-fold) than expression of *MGP-203*. To verify if expression of both transcript variants is specific only to drug resistant cell lines or can be expressed also in other cancer cell lines, we investigated expression in breast cancer cell line T47D that is known to present *MGP* expression at high level [38]. Similar to results in drug resistant cell lines, we also observed increased expression of both transcript variants, with higher level of MGP-201, in T47D cell line as well (about 2.5-fold). We have also compared expression of both transcripts between A2780TR2 and T47D cell lines and we discovered that expression any of them was about two times higher in T47D cell line. Thus, proportion between both transcript expression seems to be a general feature of cancer cells. 

Immunofluorescence analysis confirmed elevated expression of MGP protein in investigated ovarian cancer cell lines, which indicates that MGP is equally expressed in all cells. Since this method does not allow distinguishing protein isoforms, we evaluated Western blot analysis to compare the protein expression levels of MGP between investigated cell lines. The differences in expression of two MGP variants were also confirmed at the protein level. In breast cancer cell line, both variants of MGP proteins was clearly visible although isoform with molecular weight of 15.32 kDa seems to be expressed at much higher level. These results correlate with expression on transcript level and additionally indicate that the antibody used for the experiment is able to recognize both protein isoforms. In ovarian cancer cells only one form with molecular weight of 15.32 kDa was noted. This form was overexpressed in cell lines resistant to PAC and TOP. Since we previously observed that COL3A1, LUM [14,15] and MYOT (under review) are secreted to cell culture media in drug resistant ovarian cancer cell lines, we were interested whether MGP can also be present in the culture medium. Indeed, we have noticed that MGP protein was present in culture media from investigated drug resistant cell lines. As opposed to cell lysates, the MGP isoform with a mass of 12.35 kDa was observed in the corresponding media.

In all investigated cell lines, we could also observe additional bands with molecular mass about 110 kDa. We were not sure whether these bands are specific, but we paid attention to them since their intensity clearly correlated with MGP transcript and protein level in investigated cell lines. This result is difficult to interpret because molecular mass of MGP is about 10-fold lower. On the other hand, clear correlation with MGP expression suggests two possibilities. There could be another protein with coordinate expression and recognized by the same antibody or a different formation of multimeric MGP composed of several subunits. Because a Western blot experiment is conducted under denaturating and reductive condition, these subunits should bind to each other by covalent bonding, however this is only a hypothesis.

To our knowledge, this is the first report about MGP expression in drug resistant cell lines. We undertook a comprehensive literature search and did not find any research concerning MGP expression in cancer cells resistant to cytotoxic drugs. However, different levels of MGP transcripts were observed in different human and rat glioblastoma cell lines [26,43] and MGP protein released to culture medium was observed in glioblastoma cell line as well [26]. These findings confirm that MGP is expressed in different neoplastic cell lines and can be secreted to cell culture medium. However, in those studies, the primers specific to transcript variants was not considered and the protein level was assessed by the ELISA test. Therefore, we could not compare or refer our results to other research and our result need further validation and explanation. However, different localization of MGP-201 and MGP-203 suggest their different role in drug resistance.

The general question is what is the role of MGP in drug resistance. There can be three different possibilities taken under consideration. The first one presumes that MGP molecules, found both intracellularly and extracellularly, can bind PAC and TOP molecules limiting their availability for cancer cells. Direct binding of PAC, DOX and VIN by ECM compounds has been reported previously by others [5]. The potential role of MGP in PAC and TOP binding require further investigation using other methods.

The second possibility is a limited drug diffusion. It is assumed that MGP does not play a role as a single protein in this action but rather in coordination with other ECM components. Other research describes the increased expression of ECM, especially collagens that limit drug diffusion in cancer tissue and in in vitro study [5,7,44,45]. On the other hand, all investigated cell lines expressed different ECM molecules including different collagens as we reported previously [37]. Even in 2D cell culture condition we observed that COL3A1 was present extracellularly forming structure similar to a spiderweb [14]. Since it was already proved that MGP may incorporate into fibronectin multimers [27], e it is also possible that this small molecule may place between collagen fibers and in this way limit drug delivery to the cancer cells. To prove this hypothesis, further analysis with the use of 3D cell culture condition should be applied.

The third possibility of MGP in drug resistance involvement is the role of extracellular MGP in CAM-DR [10,41]. Interaction of cancer cells with their microenvironment can lead to inhibition of drug induced apoptosis. Cancer cells interact with ECM compounds mainly by integrin receptors. CAM-DR has been observed both in vivo and in vitro. The interaction of β1-integrin with ECM was proven to be responsible for resistance to DOX and melphalan in SCLC [11]. On the other hand, collagen type I is considered as involved in inducing chemoresistance by upregulating microtubule associated protein tau in paclitaxel resistant ovarian cancer cell lines [46]. In in vitro study with A2780 ovarian cancer cell line revealed that those cells cultured on COL6A3 coated dishes were more resistant to CIS [12]. CAM-DR can be induced not only by collagen molecules but was also observed for other ECM compounds like fibronectin and laminin. In this case, pancreatic cancer cells became resistant to DOX, CIS and 5-fluorouracil (5-FU) [47] when growing on fibronectin and laminin and breast cancer cells were more resistant to PAC on surfaces coated with collagen type I [48]. This might indicate a coordinated response between tumor cells and its microenvironment to protect cancer cell against drugs and to facilitate malignancy progress.

**Figure 7:**
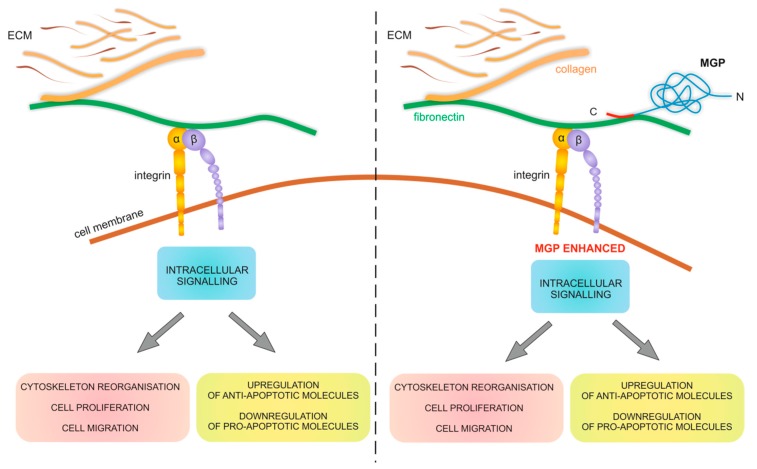
Schematic drawing of interaction between cell and ECM mediated by fibronectin and MGP. MGP augmented binding of integrins to ECM compounds activates intracellular signaling that results in: (1) cytoskeleton reorganization leading to increased cell proliferation and migration; and (2) upregulation of anti-apoptotic molecules and downregulation of pro-apoptotic molecules that inhibits drug-induced apoptosis.

The role of secretory MGP in CAM-DR seems to be related rather to its interaction with other ECM proteins. It has already been proven that MGP binds to fibronectin and vitronectin and augments cell adhesion and spreading of cancer cells. The protein itself has no adhesive activity and its role is to alter the ability of cells to bind fibronectin [27]. On one side, fibronectin interacts with cells via integrins which connect to the actin cytoskeleton and, on the other, with ECM constituents such as collagens and laminins. MGP presence augments this effect and this property is related to migration-promoting activity that was demonstrated for glioblastoma [26] or osteosarcoma cells [28]. Since the interaction of cells with fibronectin lead to increased resistance to different cytotoxic drugs (CAM-DR) and MGP augments this interaction, we have formulated a hypothesis that MGP–fibronectin interaction can intensify CAM-DR in cancer cells (Figure 7).

To further prove the significance of MGP expression in PAC and TOP resistance, we performed experiments with short time exposure of cancer cells to investigated drugs. Most research concerning drug resistance is based on “established” mechanism of resistance to cytotoxic agents and comparison of sensitive and resistant pairs of cell lines. It is difficult to find papers that demonstrate the response of cancer cells to cytotoxic drugs during first hours of treatment. Recently, we have published a few papers describing the expression of genes in response to PAC [49], TOP [50,51] and CIS [51] treatment in drug sensitive ovarian cancer cell lines. In the present study, we could observe a dose- and time-dependent increase of *MGP* mRNA in response to PAC and TOP treatment. The increased expression during the first days after contact with cytotoxic drugs confirms that MGP can indeed be involved in PAC and TOP resistance. Along with our previous results [49,50,51], this finding indicates that the genes expressed at the beginning of the treatment, a short time after drug exposure, are further expressed in established drug resistant cell lines (at much higher level). Thus, “first response” can be an indicator of “established” response after long time to cytotoxic drug exposure.

Previously, upregulation of MGP expression in ovarian cancer tissue was noted by Hough et al. but only at transcript level [52]. To verify MGP expression on protein levels in real cancer patients, we performed immunohistochemistry using paraffin sections. All of the ovarian cancer subtypes cells were positive for MGP. However, the intensity of immunohistochemical staining in our study was different and dependent on the type of ovarian cancer (endometrioid, serous and mucinous ovarian cancer). The weaker expression was present in serous and mucinous carcinoma (mild IRS score) when compared to endometrioid adenocarcinoma where moderate to strong signal was detected. Additionally, in the endometrioid adenocarcinoma we could observe subpopulations of cancer cells with different levels of MGP protein expression (high and weak/moderate).

Similar observations were made by other researchers. In samples with cervical squamous cell carcinoma (SCC), clear cytoplasmic MGP staining was seen in the tumor cells but in cells seen at the borders of the tumor fields the staining was more intense. Moreover, they could also observe a clear strong staining for individual tumor cells of SCC. In the same study, they could also observe elevated MGP expression only in the lower layers of epithelium of high grade cervical intraepithelial neoplasia (CIN) lesions [53]. The analysis and interpretation of immunohistochemical stainings must be done with a great caution since the MGP expression level of individual cancer cells can vary even within a single cancer cell nest.

The expression of MGP was noted in some other cancers. In line with our findings concerning ovarian cancer, increased levels of MGP were found in glioblastomas, breast, cervical and skin cancer where positive correlation with tumor progression and survival was observed [26,54,55,56]. Yoshimura K et al. proved the correlation between mRNA levels of MGP and a poor prognosis of breast cancer patients [24]. The increased expression of MGP correlated with increased migration of tumor cells in vivo and increased spreading and metastases formation in mouse model of osteosarcoma [28]. Migratory activity of cells with elevated levels of MGP was also observed in vitro. Among the glioma cell lines used in the experiment, those with knocked down MGP gene presented decreased cell migration ability when compared to cells overexpressing MGP [26]. Additionally, the role of circulating levels of MGP was described for osteosarcoma patients who revealed a significant increase of this protein at the time of diagnosis and then developed lung metastases [28].

The role of MGP produced by cancer cells remains still not clear and its main function seems to be limited mainly to tumor environment. Therefore, the role of MGP in tumor progression should be considered from the stromal point of view where MGP–ECM interactions could trigger changes and rearrangements of tumor microenvironment.

## 4. Materials and Methods

### 4.1. Reagents and Antibodies

PAC and TOP were obtained from Sigma (St. Louis, MO, USA). RPMI-1640 and MEM medium, fetal bovine serum, antibiotic–antimycotic solution, and L-glutamine were also purchased from Sigma (St. Louis, MO, USA). Rabbit polyclonal anti-MGP Ab was obtained from Proteintech (Manchester, UK). Donkey anti-goat horseradish peroxidase- (HRP) conjugated Ab was purchased from Santa Cruz Biotechnology (Santa Cruz, CA, USA). The fluorescent secondary antibody, Alexa Fluor®488 Donkey Anti-Rabbit IgG, was obtained from Jackson ImmunoResearch Laboratories (Cambridgeshire, UK). The mounting medium with DAPI was obtained from Santa Cruz Biotechnology (Santa Cruz, CA, US).

### 4.2. Cell Lines and Cell Culture

A2780 human epithelial ovarian cancer cell line was obtained from ATCC (American Type Culture Collection, Manassas, VA, USA) and drug-resistant derivatives of A2780 cells were developed by exposing them to cyclic drug treatment. Paclitaxel resistant subline (A2780PR1) and topotecan resistant sublines (A2780TR1 and A2780TR2) were developed by subjecting A2780 cells to incremental doses of paclitaxel or topotecan. The final concentrations used for selecting the resistant cells were 300 ng/mL of PAC and 24 ng/mL of TOP. The increase in resistance according to parental drug sensitive cell lines were as follow: 146-fold for A2780PR1 vs. A2780; 59.6-fold for A2780TR1 vs. A2780; and 48.5-fold for A2780TR2 vs. A2780 as described previously [3]. All of the cell lines were maintained as monolayers in the MEM medium supplemented with 10% fetal bovine serum, 2 pM L-glutamine, penicillin (100 units/mL), streptomycin (100 units/mL) and amphotericin B (25 μg/mL) at 37 °C in a 5% CO_2_ atmosphere.

### 4.3. Examination of Gene Expression Using QPCR

Total RNA was extracted from A2780 and drug-resistant cell lines using the GeneMATRIX Universal RNA purification kit (EURx Ltd. Gdansk, Poland) as described by the manufacturer’s protocol. Reverse transcription was performed using M-MLV reverse transcriptase (Invitrogen by Thermo Fisher Scientific, Waltham, MA, USA) and a thermal cycler (Veriti 96-well Thermal Cycler) according the manufacturer’s instructions. Two micrograms of RNA were used for cDNA synthesis. Real-time PCR was performed using the 7900HT Fast Real-Time PCR System (Applied Biosystems, Foster City, CA, USA), Maxima SYBR Green/ROX qPCR Master Mix (Thermo Fisher Scientific, Waltham, MA, USA) and the sequence-specific primers that are indicated in Table 1. Glyceraldehyde-3-phosphate dehydrogenase (*GADPH*), *β-actin*, hypoxanthine-guanine phosphoribosyltransferase 1 (*HRPT1*) and beta-2-microglobulin (*β2M*) served as internal control housekeeping genes to normalize the PCRs (geometric mean) for the gene expressions being analyzed. Reactions were performed in a total volume of 24 μL, including 12.5 μL of Maxima SYBR Green/ROX qPCR Master Mix (Thermo Fisher Scientific, Waltham, MA, USA), 1 μL of each primer (Oligo, Warsaw, Poland) (Table 1), 9.5 μL of water and 1 μL of the reverse-transcribed cDNA template. One RNA sample from each preparation was processed without the RT-reaction to provide a negative control in the subsequent PCR reaction. After amplification, melting curves were used to determine the specificity of the gene products, which was confirmed by running the PCR products on 3% agarose gel. Gene expressions were analyzed using the relative quantification (RQ) method. The RQ method estimates the differences in gene expression against a calibrator (drug-sensitive line) (RQ of the calibrator = 1). The drug-sensitive A2780 cell line was used as the calibrator. The analysis was conducted using the following standard formula: RQ = 2^−ΔΔCt^ (where ΔΔCt = ΔCt of the sample (drug-resistant line) − ΔCt of the calibrator (drug sensitive line)). The graphs were plotted using Sigma Plot.

### 4.4. Protein Isolation from Cell Culture and Media

Whole cell lysates from drug-sensitive and drug-resistant ovarian cancer cells (1 × 10^6^ cells/20 μL lysis buffer) were lysed using containing protease inhibitor cocktail (Roche Diagnostics GmbH, Mannheim, Germany) for 10 min on ice. The lysates were centrifuged at 8000× *g* for 10 min at 4 °C, and protein concentration was quantified using the Bradford protein assay system (Bio-Rad Laboratories, Hemel Hempstead, UK). The isolation of proteins from media was preceded by culturing cells in serum-free media for 72 h. After that, the media was centrifuged at 15,000 rpm for 30 min at RT. Then, the supernatants were transferred to Amicon Ultra-15 3K centrifuge filter devices and centrifuged according the manufacturer’s protocols (40 min, 4000× *g*, RT, swinging-bucket rotor).

### 4.5. SDS-PAGE and Western Blot Analysis of MGP

Protein samples (20 μg each) were resuspended in 4× loading buffer (Bio-Rad Laboratories, Hemel Hempstead, UK) and incubated at RT for 20 min. Next, they were loaded onto a four 20% mini-PROTEAN^®^ TGX™ precast gels using the SDS-PAGE technique and finally transferred onto nitrocellulose membrane. This step was followed by blocking in 5% milk in TBS/Tween (0.1 M Tris-HCl, 0.15 M NaCl, 0.1% Tween 20) and immunodetection using rabbit anti-MGP Ab at 1:1000 dilution, and the appropriate HRP-conjugated secondary Ab. Membrane was washed in TBST and develop during a chemiluminescence detection system (ECL, Femto Super Signal Reagent) and Hyperfilm ECL (GE Healthcare, Buckinghamshire, UK). To normalize protein loading in the lanes, the membranes were stripped and reblotted with rabbit anti-GADPH Ab, from Santa Cruz Biotechnology, at a 1:1000 dilution and goat anti-rabbit HRP-conjugated Ab. The relative density of MGP to that of GADPH was analyzed with ImageJ Java-based image processing program developed at the National Institutes of Health (University of Wisconsin, Madison, WI, USA).

### 4.6. Immunofluorescence Analysis

The cells were cultured on microscopic glass slides and grown to a near-confluent state. Immunofluorescence analysis was conducted following the previously established protocol [15]. Briefly, the cells were fixed with 4% PFA, permeabilized in ice-cold acetone/methanol (1:1) and then washed with PBS. After blocking with 3% BSA the cells were incubated with the anti-MGP primary antibody (rabbit polyclonal anti-MGP antibody, 1:100 Proteintech, Manchester, UK) for 2 h at room temperature. Next, the cells were washed with PBS and incubated with the secondary antibody for 1 h at room temperature (Alexa Fluor®488 Donkey Anti-Rabbit IgG, Jackson ImmunoResearch Laboratories, Cambridgeshire, UK). Afterwards, the cells were washed with PBS and sealed with DAPI-containing mounting medium. The cells were imaged for MGP expression analysis using fluorescence microscope (Zeiss Axio-Imager.Z1, Oberkochen, Germany). The pseudo-color representation of fluorescence intensity was assessed for DAPI at 365 nm excitation and 420 nm emission wavelengths (blue) and for Alexa Fluor^®^488 at 470 nm excitation and 525 nm emission wavelengths (green).

### 4.7. Incubation of Cells with PAC and TOP in Time-Course experiment

The drug-sensitive A2780 cells were seeded into 6-well plates at 0.5 × 10^6^ in 1 mL of medium per well. Cells were treated with low concentrations of PAC (20 and 25 ng/mL) or TOP (10 and 20 ng/mL) for 24, 48 and 72 h. After each period of incubation, cells were harvested and the RNA isolation was conducted.

### 4.8. Immunohistochemistry

Immunohistochemical staining was conducted for formalin-fixed, paraffin embedded human ovarian carcinomas lesions. The analysis of MGP expression was performed using the polymer-based immunohistochemical (IHC) technique [57] using an EnVision^TM^ HRP-polymer anti-mouse/rabbit IHC Kit (Dako REAL EnVision^TM^ Detection System peroxidase/DAB+, Rabbit/Mouse, Dako, Glostrup, Denmark) according to the manufacturer’s guidelines. Briefly, prior to immunhistochemical staining, the slides were dewaxed and hydrated. The blocking of the activity of endogenous peroxidise with 1% H_2_O_2_ for 30 min was followed by incubation with the primary antibody at 4 °C/overnight (rabbit polyclonal anti-MGP antibody, 1:100 Proteintech, Manchester, UK). Then, the incubation with EnVision Detection System for 30 min at room temperature was followed by hematoxylin counterstaining. Finally, the sections were dehydrated, mounted and examined under the optical Olympus BH-2 microscope coupled to a digital camera. Color microscope images were recorded using LUCIA Image 5.0 computer software (Nikon, Tokyo, Japan).

The expression of MGP was established by mean proportion of immunopositive cancer cells as well as surrounding stroma, for 10 microscope fields at magnification of 400×. For each specimen, the immunoreactivity score (IRS) was derived by multiplying intensity score by distribution score. Intensity for cancer cell staining was scored as negative (0), mild (1+), moderate (2+), or strong (3+). The distribution of staining of cancer cells was scored as 0 (no positive cells), 1+ (<10% of cells staining), 2+ (10–50% of cells staining), 3+ (51–80% of cells staining) or 4+ (>80% of positive cells). IRS score was interpreted as negative (IRS 0-1), mild (IRS 2-3), moderate (IRS 4-8) or strongly positive (IRS 9-12). Staining intensity was also determined in stromal tissues adjacent to cancer cells. Intensity of stroma staining was scored as negative (0), mild (1+), moderate (2+), or strong (3+).

### 4.9. Statistical Analysis

Statistical analysis was performed using Microsoft Excel software. The statistical significance of the differences was determined using the Student’s *t*-test, and *p*-values of 0.05 or less were considered statistically significant.

## 5. Conclusions

In summary, to our knowledge, this is the first report about MGP expression in drug resistant cell lines and in ovarian cancer tissue at protein level. The research found elevated levels of MGP expression in PAC and TOP resistant ovarian cancer cell lines and corresponding media as well as in response to short time PAC and TOP treatment. For the first time, we have described the coordinate expression of two MGP isoforms in ovarian and breast cancer cell lines. Additionally, as secreted outside the cell, the MGP protein could be implicated in cell adhesion-mediated drug resistance (CAM-DR). Therefore, we have demonstrated that MGP is an important factor that might contribute to cancer resistance mechanism by augmenting the interaction of cells with ECM components leading to increased resistance of ovarian cancer cells to paclitaxel and topotecan.

The results of present research may improve our understanding of the role of MGP in ovarian cancer biology and help identify new therapeutic targets. 

## Figures and Tables

**Table 1:** Oligonucleotide sequences used for RQ-PCR analysis.

Transcript	Sequence (5′-3′ direction)	ENST Number http://www.ensembl.org	Product Size (bp)
MGP	CTGATCCTTCTTGCCATCCT CCATCTCTGCTGAGGGGATA	00000228938.5 00000539261.5	141 bp
MGP-201	GTGCCCAGGAATCACATGAAAG ACAGGCTTAGAGCGTTCTCG	00000228938.5	142 bp
MGP-203	AAGAGAGGATCCGAGAACGCC AGCGTTCGCAAAGTCTGTAG	00000539261.5	81 bp
GADPH	GAAGGTGAAGGTCGGAGTCA GACAAGCTTCCCGTTCTCAG	00000229239	199 bp
β-actin	TCTGGCACCACACCTTCTAC GATAGCACAGCCTGGATAGC	00000331789	169 bp
HRPT1	CTGAGGATTTGGAAAGGGTG AATCCAGCAGGTCAGCAAAG	00000298556	156 bp
β2M	CGCTACTCTCTCTTTCTGGC ATGTCGGATGGATGAAACCC	00000558401	133 bp

## Abbreviations

TOP	Topotecan
PAC	Paclitaxel
MGP	Matrix Gla Protein
ECM	Extracellular Matrix
CAM	Cell Adhesion Molecule
TR	Topotecan Resistance
PR	Paclitaxel Resistance
